# Supplementary material for: A bidentate Polycomb Repressive-Deubiquitinase complex is required for efficient activity on nucleosomes
Source: Nat Commun. 2018 Sep 26;9:3932. doi: 10.1038/s41467-018-06186-1 (PMC6158172; doi:10.1038/s41467-018-06186-1)
Supplement: Supplementary file 1 — Supplementary Information [file 41467_2018_6186_MOESM1_ESM.pdf]

Supplementary Information

A bidentate Polycomb Repressive-Deubiquitinase complex is required for efficient activity on nucleosomes.

Foglizzo et al.

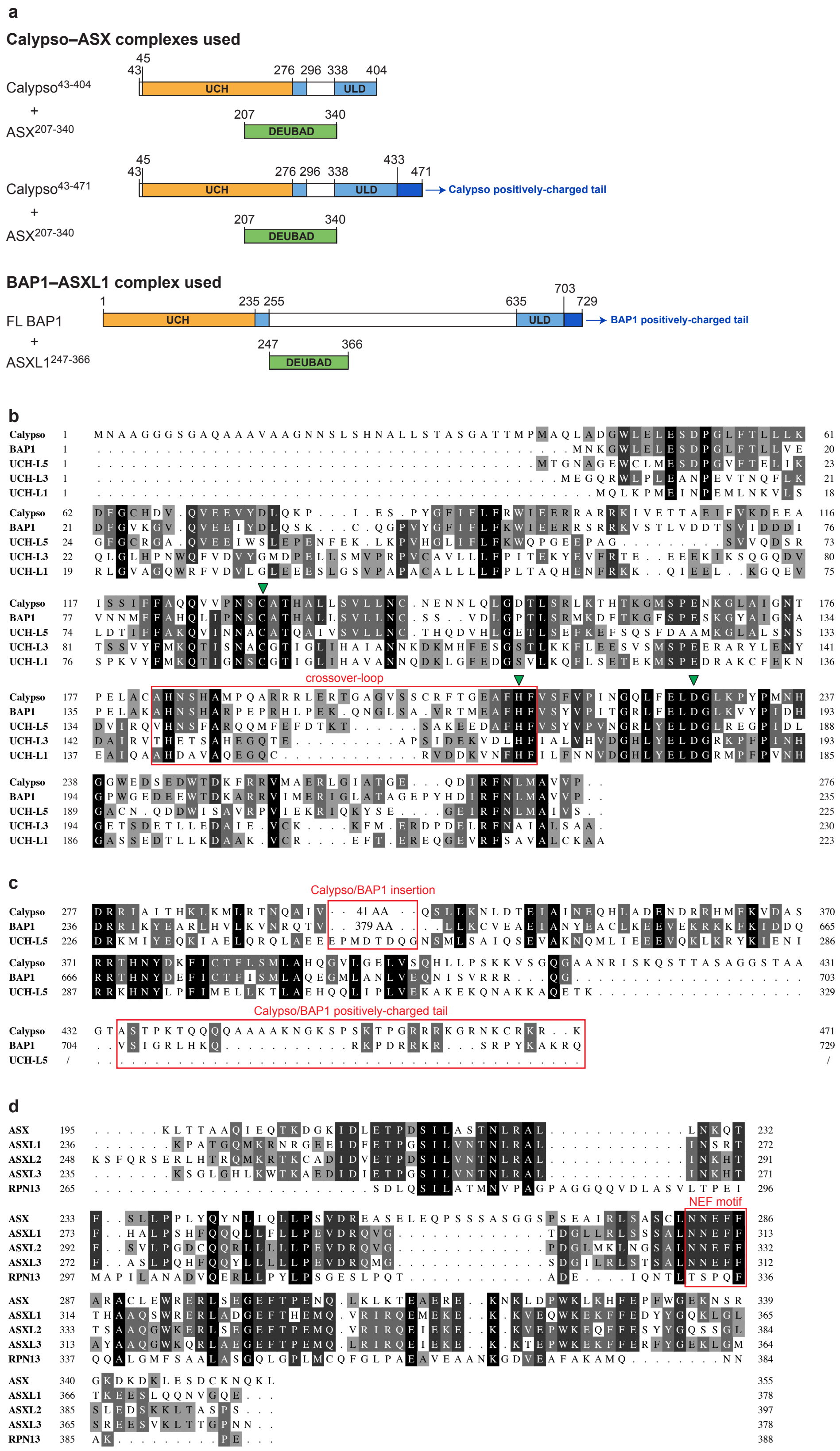

Supplementary Fig. 1| Comparison of Calypso and ASX with other family members. a, Schematic of the Calypso–ASX and BAP1–ASXL1 complexes used in this study. UCH = ubiquitin C-terminal hydrolase; ULD = UCH37-like domain; FL = full-length; ASX = additional sex combs; ASXL1 = ASX-like 1. **b**, Multiple sequence

alignment of the UCH domains in Calypso, BAP1, UCH-L5, UCH-L3 and UCH-L1. Identity between the proteins is indicated with black shading; similarity with grey shading. The crossover-loop is highlighted with a red box; green arrows indicate the residues forming the active site triad. **c**, Multiple sequence alignment of the ULD domains in Calypso, BAP1 and UCH-L5. Identity and similarity between the proteins are indicated as in **b**. The Calypso/BAP1 insertion and the C-terminal positively-charged tail are highlighted with red boxes. **d**, Multiple sequence alignment of the Deubad domains in ASX, ASXL1, ASXL2, ASXL3 and Rpn13. Identity and similarity between the proteins are indicated as described in **b**. The NEF region is highlighted with a red box. Deubad = deubiquitinase adaptor domain; ASXL = ASX-like.

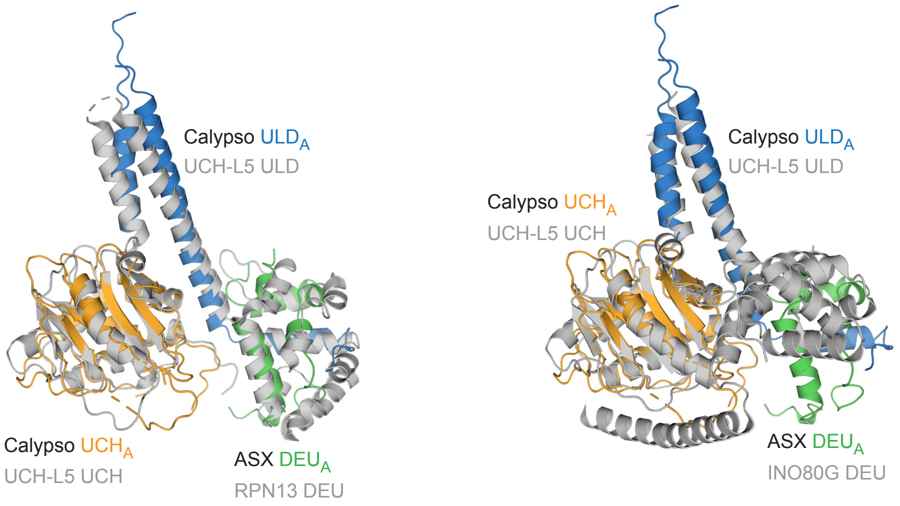

Supplementary Fig. 2| Comparison of the Calypso–ASX complex with the activated and inhibited UCH-L5 complexes. Overlay of Calypso–ASX onto the structures of the UCH-L5–RPN13 (PDB code: 4UEM) (*left*) and UCH-L5–INO80G (PDB code: 4UF5) (*right*) complexes (ref. [1]). The UCH and ULD domains of Calypso, and the Deubad domain of ASX, are shown in cartoon and colored as described in **Fig. 1b**. The corresponding domains of UCH-L5 and RPN13/INO80G are colored grey. Chain B of the Calypso–ASX structure has been removed for clarity. UCH = ubiquitin C-terminal hydrolase; ULD = UCH37-like domain; Deubad (DEU) = deubiquitinase adaptor domain; ASX = additional sex combs.

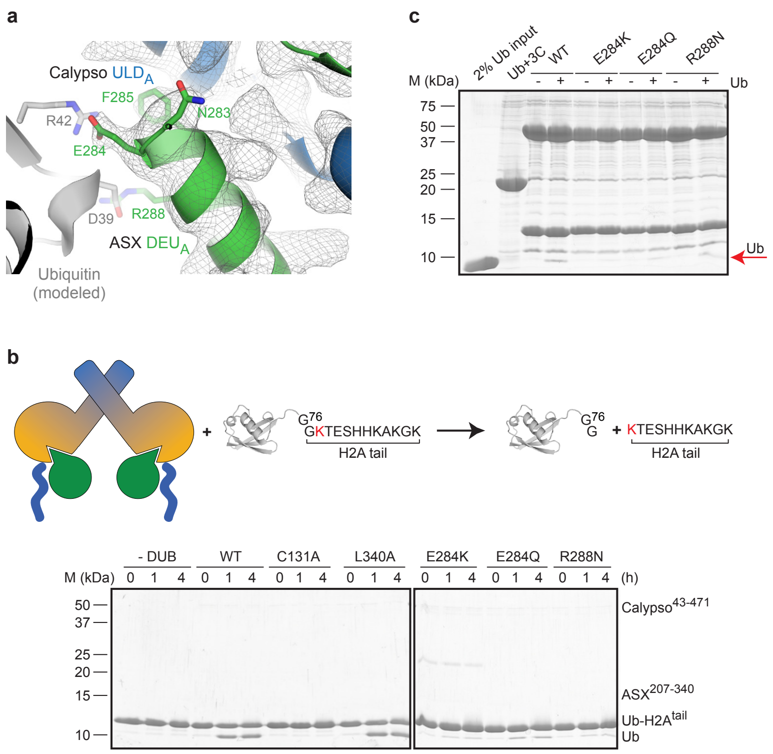

Supplementary Fig. 3| The *Drosophila* PR-DUB complex as a model for understanding the impact of cancer-derived missense mutations in the human PR-DUB. **a**, A 2Fo-Fc electron density map (contoured at 1 σ level) around the NEF region of the ASX Deubad domain. Residues in the NEF-motif of ASX, Arg288, and the putative interacting amino acids in the modeled ubiquitin are shown as sticks. ULD = UCH37-like domain; Deubad (DEU) = deubiquitinase adaptor domain; ASX = additional sex combs. **b**, Schematic representation of the Ubiquitin-H2A^{tail} hydrolysis assays performed in this study (*top*). The additional glycine residue following ubiquitin Gly76 provides a linker between the C-terminus of ubiquitin and the N-terminus of the Histone 2A tail. Deubiquitination assays comparing the ability of wild-type and mutant Calypso–ASX complexes to remove ubiquitin from Ubiquitin-H2A^{tail} (*bottom*). Reactions were incubated at 37°C for 4 h; samples were analysed using 14-20% SDS-PAGE gradient gels and visualised by staining with Coomassie Blue. C131A refers to a mutation in the active site Cysteine of Calypso. DUB = deubiquitinating enzyme; WT = wild-type; Ub = Ubiquitin; H2A = Histone 2A. **c**, His_6-fused wild-type and mutants Calypso–ASX complexes were bound to Ni^{2+}-NTA beads and tested in pull-down experiments with purified ubiquitin. The extent of ubiquitin binding was detected by Coomassie Blue staining.

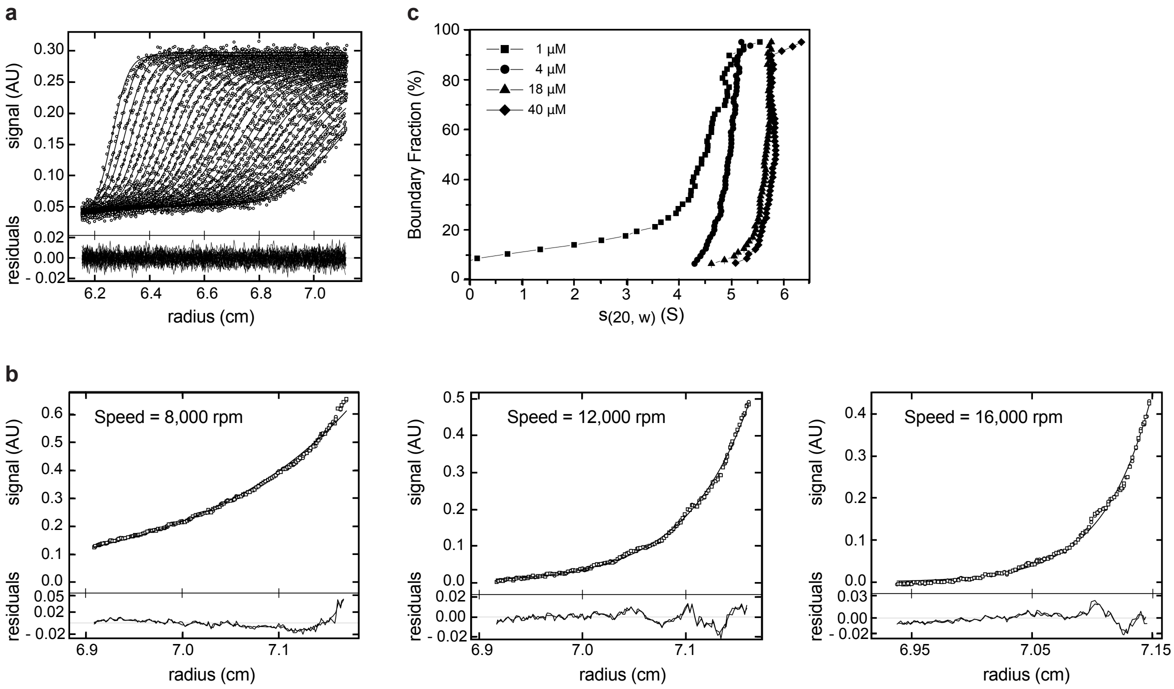

Supplementary Fig. 4| Characterisation of the Calypso–ASX oligomeric state by analytical ultracentrifugation. **a**, Raw sedimentation velocity data and residuals, corresponding to data shown in **Fig. 3b**, for Calypso–ASX at 40 µM. **b**, Sedimentation equilibrium data at three speeds, fitted to a single species model with a fitted mass of 112.9 kDa (+/- 0.3 kDa). Each panel shows two data sets, taken 1 hour apart, to demonstrate that the system had come to equilibrium. The global reduced chi-squared for the fit was 2.35 (n=857). **c**, van Holde-Weischet analysis of Calypso–ASX at concentrations of 1 µM (squares), 4 µM (circles), 18 µM (triangles) and 40 µM (diamonds).

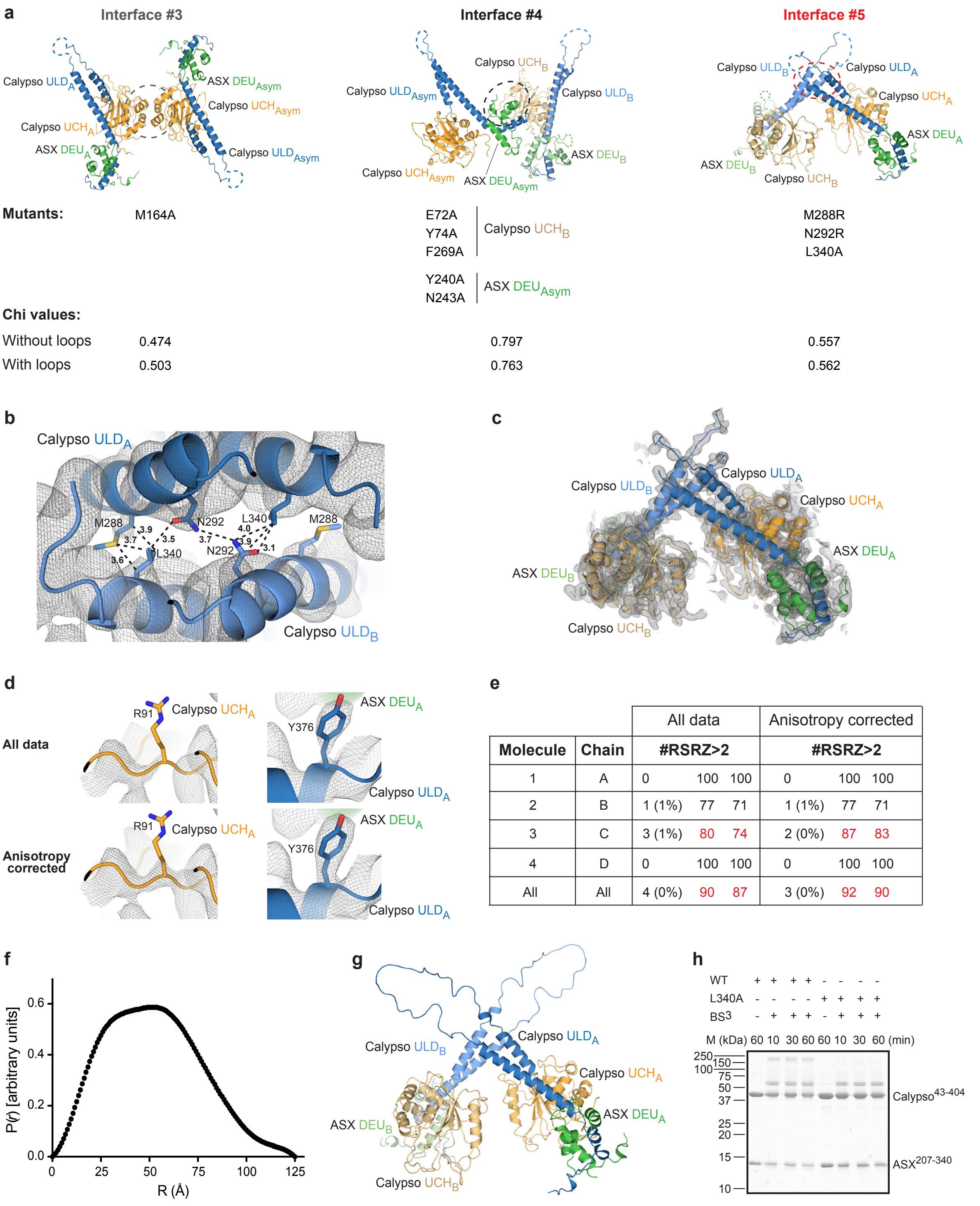

Molecule	Chain	All data #RSRZ>2			Anisotropy corrected #RSRZ>2		
1	A	0	100	100	0	100	100
2	B	1 (1%)	77	71	1 (1%)	77	71
3	C	3 (1%)	80	74	2 (0%)	87	83
4	D	0	100	100	0	100	100
All	All	4 (0%)	90	87	3 (0%)	92	90

Supplementary Fig. 5| Structural, biophysical and biochemical characterisation of the 2:2 Calypso–ASX oligomer. a, Schematic of the possible hetero-tetrameric interfaces identified by PISA (ref. [2]), with the designed mutant(s) and the Chi values obtained from SEC-SAXS experiments listed below. UCH = ubiquitin C-terminal hydrolase; ULD = UCH37-like domain; Deubad (DEU) = deubiquitinase adaptor domain; ASX = additional sex combs. **b,** A composite omit map (contoured at 1 σ level) of the Calypso dimer interface, showing the packing of the two coiled-coil hairpins. Residues involved in dimerisation are shown in stick form. Main- and side-

chain contacts between residues are indicated by dashed lines, with distance measurements (numbers) shown in Å. **c**, A 2Fo-Fc electron density map (contoured at 1 σ level) of the Calypso–ASX crystal structure. The structure is shown in cartoon representation and colored as described in **Fig. 1b**. **d**, Composite omit maps (contoured at 1 σ level) for the full (*top*) and anisotropy corrected (*bottom*) datasets, showing regions in the structure that were improved after anisotropy correction. **e**, Table showing the number (and percentage) of RSRZ outliers, followed by percentile scores relative to all X-ray entries and entries with similar resolution. Percentile scores that improved after anisotropy correction are highlighted in red. **f**, iInteratomic distance distributions of the Calypso–ASX complex. **g**, Structure of the Calypso–ASX complex used for SAXS experiments, with loops that were not defined by electron density modeled. The structure is shown in cartoon representation and colored as described in **Fig. 1b**. **h**, Assays comparing the appearance of cross-linked species in wild-type and L340A Calypso–ASX complexes over time. Samples were resolved by reducing SDS-PAGE gel and stained with Coomassie Blue. WT = wild-type; BS^3 = bis(sulfosuccinimidyl)suberate.

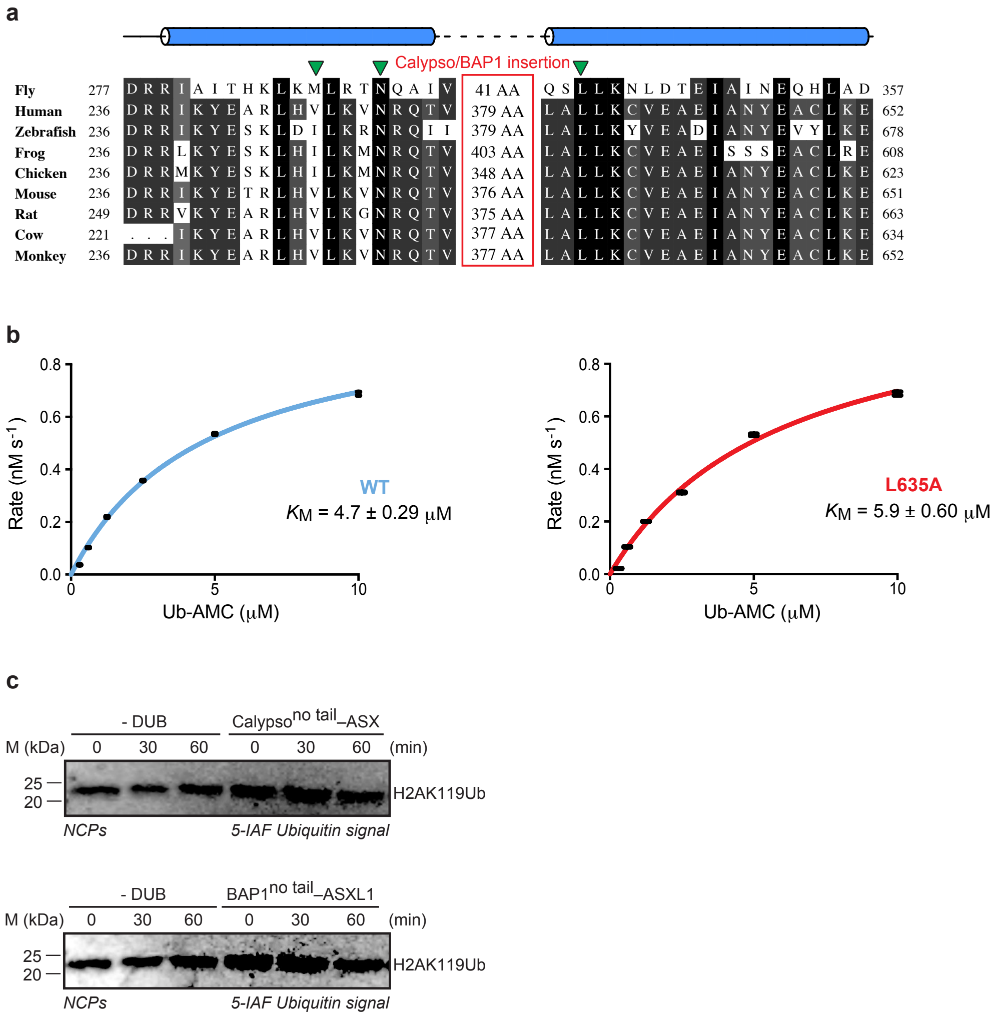

Supplementary Fig. 6| Impact of PR-DUBs oligomerisation in nucleosome recruitment and activity. a, Multiple sequence alignment of the coiled-coil hairpin across nine different BAP1/Calypso-like DUBs. Identity between the proteins is indicated with black shading; similarity with grey shading. The BAP1/Calypso insertion is highlighted with a red box; green arrows indicate the residues targeted for mutations. Secondary structure elements are indicated. **b**, Michaelis-Menten analysis of wild-type and L635A BAP1–ASXL1 proteins cleavage of Ubiquitin-AMC. Error bars indicate ±SEM (*n* = 2 independent experiments). WT = wild-type. **c**, Deubiquitination assays comparing the ability of wild-type Calypso$^{no\ tail}$–ASX (*top*) and BAP1$^{no\ tail}$–ASXL1 (*bottom*) complexes to cleave ubiquitin from H2AK119Ub nucleosomes. Assays were visualised as described in **Fig. 5b**. DUB = deubiquitinating enzyme; ASX = additional sex combs; ASXL1 = ASX-like 1.

Fig. 4e

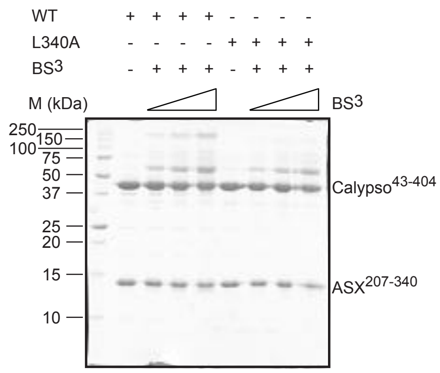

Fig. 6a

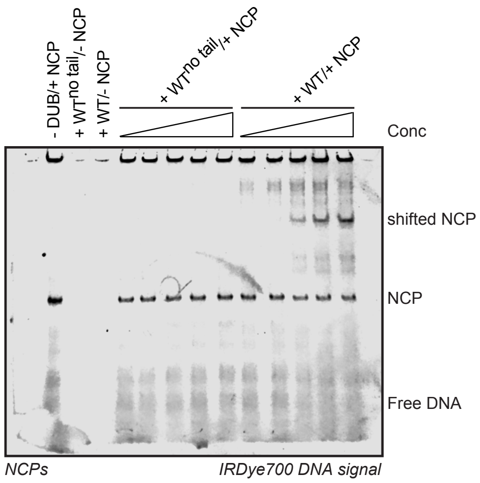

Supplementary Fig. 7| Uncropped gels for Fig. 4e and Fig. 6a.

Fig. 5b

Calypso–ASX WT and L340A assay replicates

Replicate 1

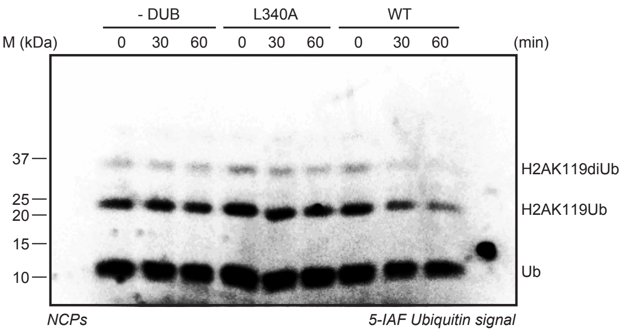

Replicate 2

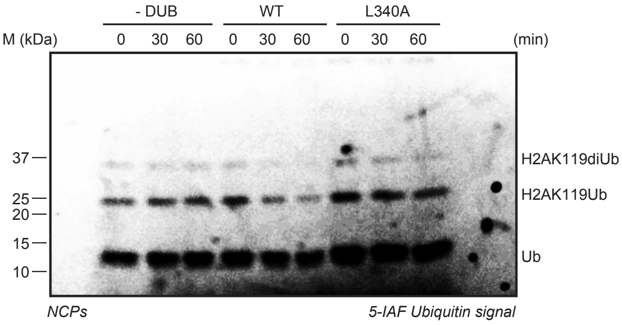

Replicate 3

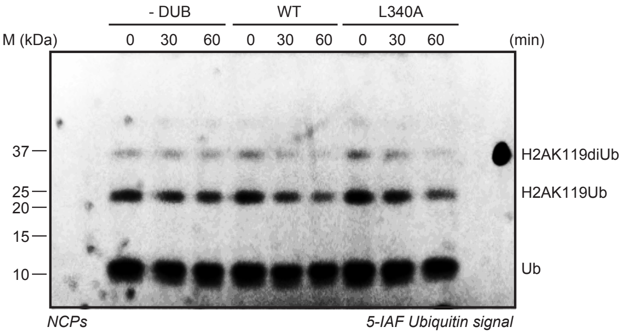

Calypso–ASX WT, M288R and N292R assay replicates

Replicate 1

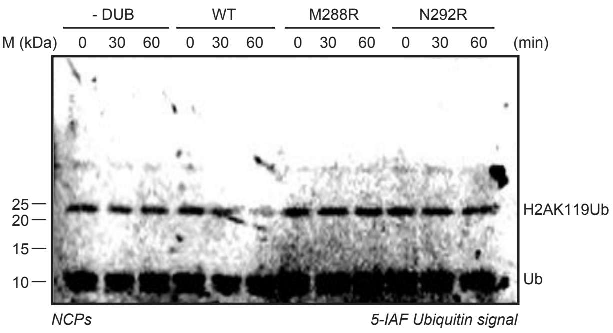

Replicate 2

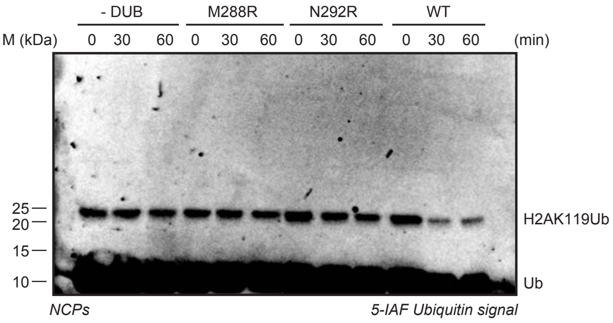

Replicate 3

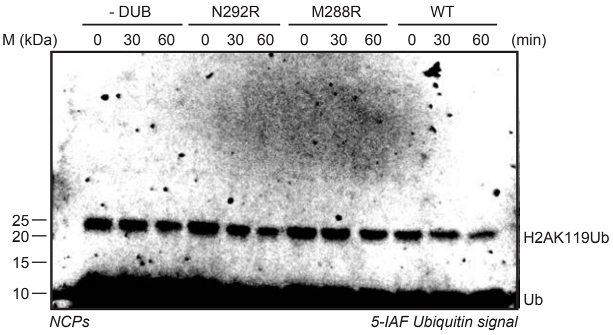

Supplementary Fig. 8| Uncropped gels for the replicate experiments shown in Fig. 5b. For each set of experiments, gels shown in **Fig. 5b** correspond to the first experimental replicate (Replicate 1).

Fig. 5c

BAP1–ASXL1 WT and L635A assay replicates

Replicate 1

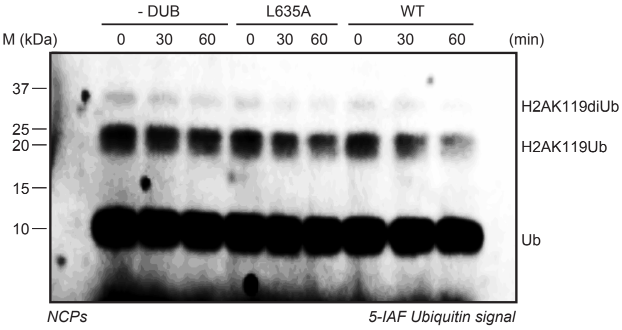

Replicate 2

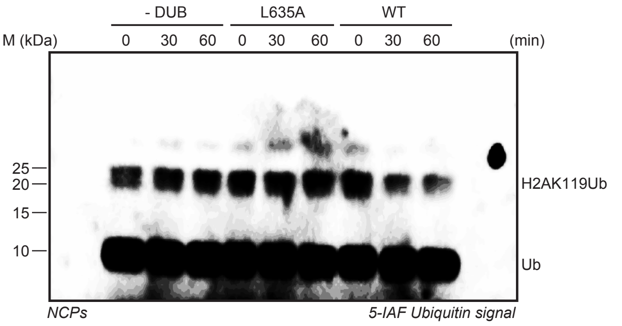

Replicate 3

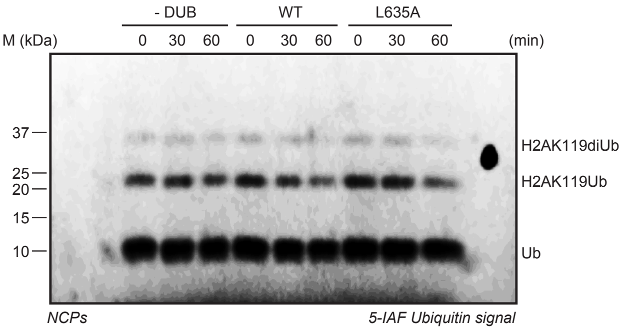

BAP1–ASXL1 WT and N251R assay replicates

Replicate 1

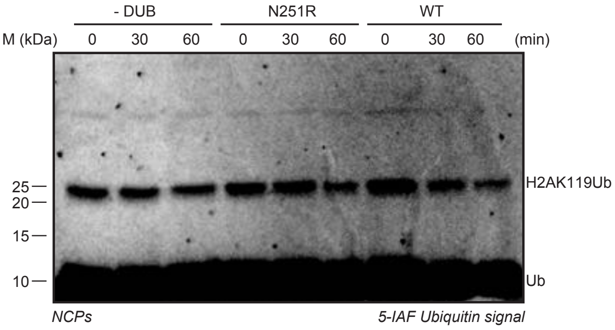

Replicate 2

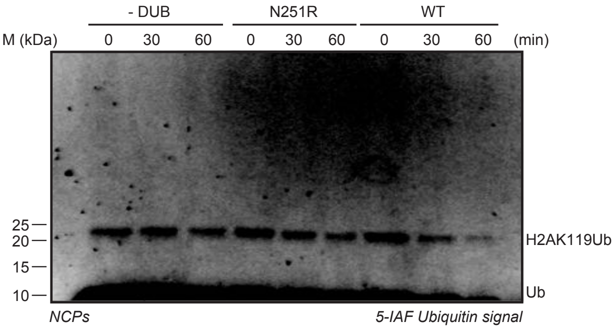

Replicate 3

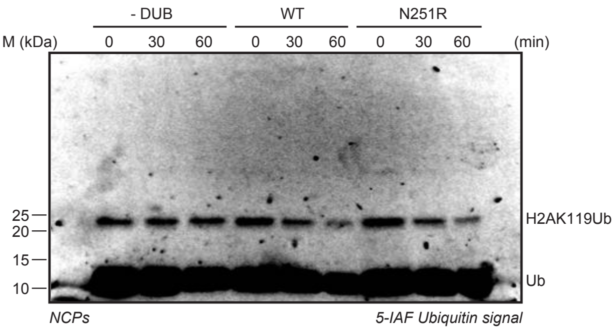

Supplementary Fig. 9| Uncropped gels for the replicate experiments shown in Fig. 5c. For each set of experiments, gels shown in **Fig. 5c** correspond to the first experimental replicate (Replicate 1).

Fig. 6b

Replicate 1

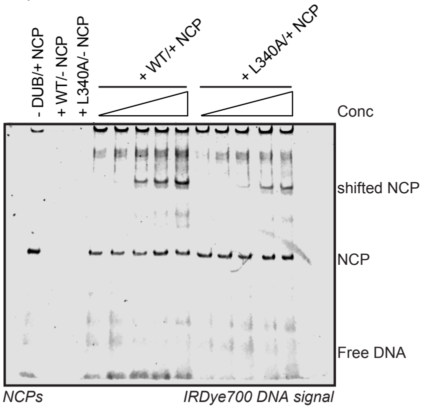

Replicate 2

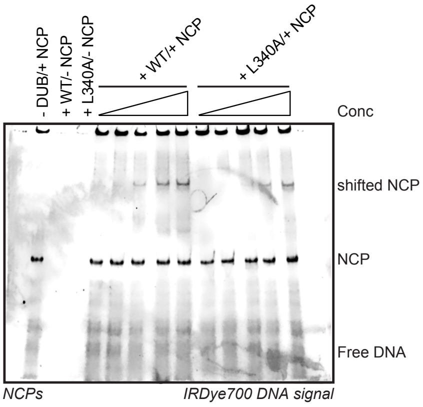

Replicate 3

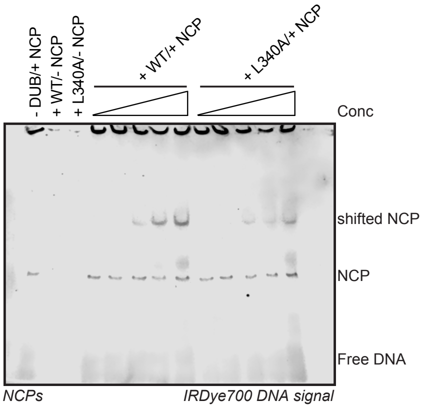

Fig. 6c

Replicate 1

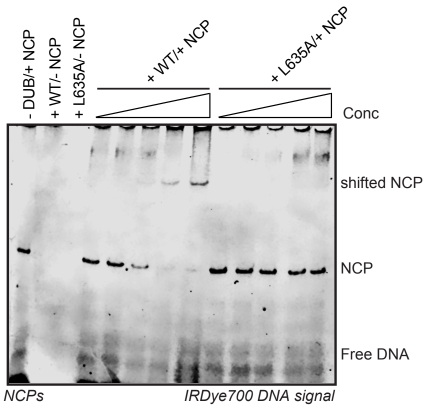

Replicate 2

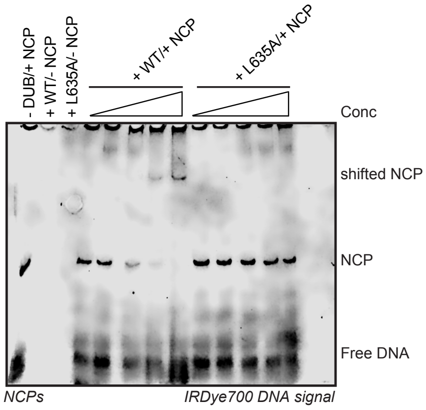

Replicate 3

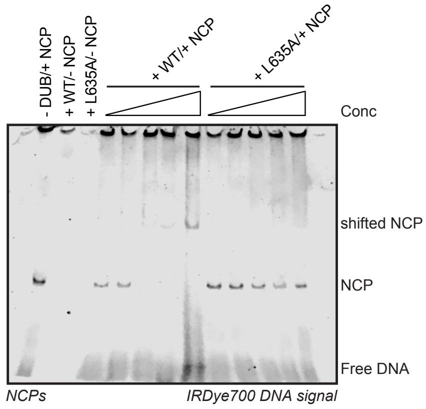

Supplementary Fig. 10| Uncropped gels for the replicate experiments shown in Fig. 6b,c. Gels shown in **Fig. 6b** and **Fig. 6c** correspond to the first experimental replicate (Replicate 1).

Supplementary Fig. 3b

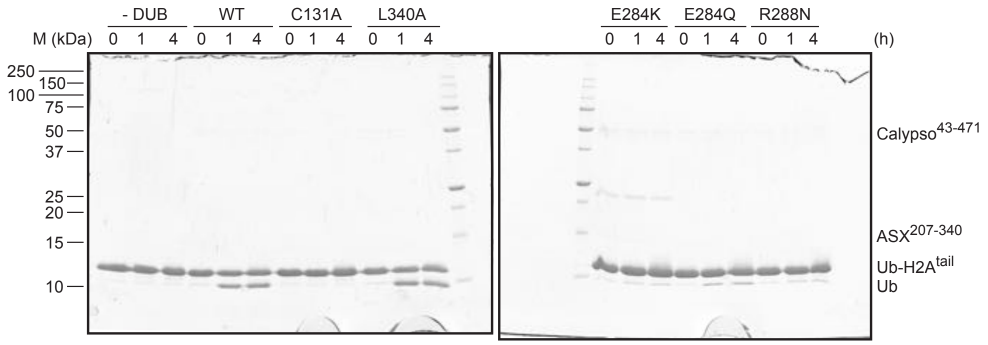

Supplementary Fig. 3c

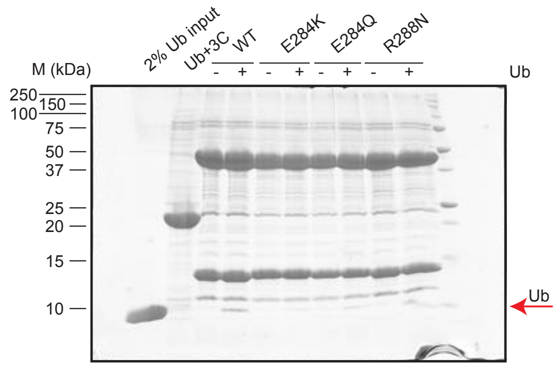

Supplementary Fig. 5e

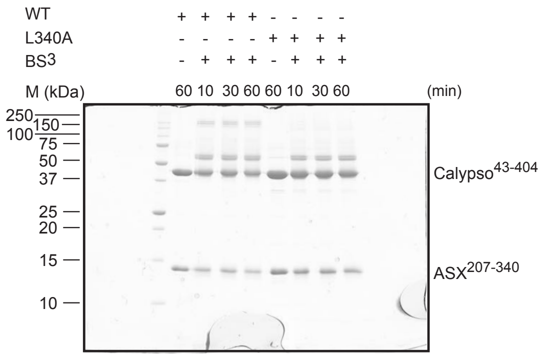

Supplementary Fig. 6c

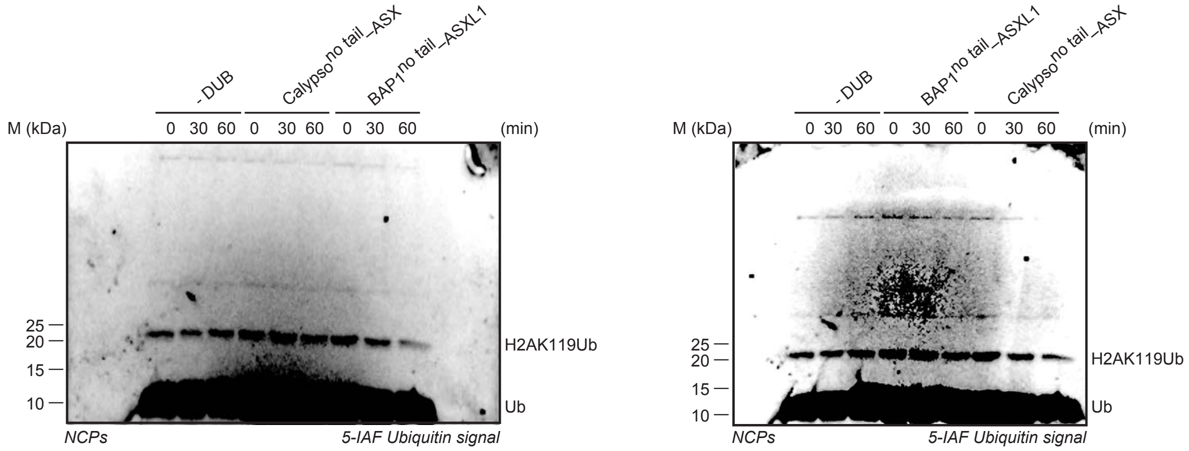

Supplementary Fig. 11| Uncropped gels for Supplementary Figures.

Supplementary Table 1. X-ray data collection and refinement statistics

	Calypso–ASX complex (PDB: 6CGA)
**Data collection**	
Space group	$I12_1$
Cell dimensions	
a, *b*, *c* (Å)	150.17, 65.63, 162.76
α, β, γ (°)	90, 114.35, 90
Resolution (Å)	43.67-3.50 (3.83-3.50)[a]
R_{pim} (all I^+/I^-)	0.062 (0.714)
Total no. of reflections	50698 (11657)
Total no. of unique reflections	18300 (4333)
Mean I / σI	7.1 (1.4)
$CC_{1/2}$	0.998 (0.613)
Completeness (%)	98.8 (99.1)
Redundancy	2.8 (2.7)
**Refinement**	
Resolution (Å)	43.67-3.50
No. atoms	
Protein	5885
Water	0
Before anisotropic scaling	
No. reflections	18271
Completeness (%)	96.57
R_{work} / R_{free}	0.265/0.303
Average *B*-factor (Å^2)	
Protein	176.86
R.m.s. deviations	
Bond lengths (Å)	0.005
Bond angles (°)	1.06
After anisotropic scaling	
No. reflections	15404
Completeness (%)	83.1
R_{work} / R_{free}	0.244/0.294
Average *B*-factor (Å^2)	
Protein	132.17
R.m.s. deviations	
Bond lengths (Å)	0.002
Bond angles (°)	0.557

Diffraction data were obtained from one protein crystal.
R.m.s. deviations, root-mean-square deviations.
[a]Values in parentheses are for the highest-resolution shell.

Supplementary Table 2. Cancer-derived missense mutations in BAP1

Mutation ID in BAP1	# Instances	Corresponding residue in Calypso	Domain ID	References
**G4C**	1	G45	UCH	TCGA[a], 2014; TCGA, unpublished
**L8R**	1	L49	UCH	DFCI[b], 2015
**S10N/T**	3	S51	UCH	TCGA, 2013; DFCI, 2016; TCGA, unpublished
**P12T/S**	2	P53	UCH	JHU[c], 2014; MSK[d], 2017
**G13D/V**	2	G54	UCH	Novartis/Broad, 2012; MSKCC[d], 2017
**L14F**	2	L55	UCH	MSKCC, 2017
**F15V**	1	F56	UCH	DFCI/MSKCC, 2014
**L17P**	1	L58	UCH	TCGA, 2013; TCGA, unpublished
**V19M**	1	L60	UCH	Novartis/Broad, 2012
**E20D**	1	K61	UCH	MSKCC, 2017
**V24G/D**	2	C65	UCH	BGI[e], 2012; METABRIC[f], 2012; METABRIC, 2016
**E30K**	1	E71	UCH	TCGA, unpublished
**E31K/V/A/G/D**	8	E72	UCH	Novartis/Broad, 2012; TCGA, 2013; TCGA, 2014; U Tokyo[g], 2013; MSKCC, 2014; TCGA, unpublished
**Y33D/C**	2	Y74	UCH	Novartis/Broad, 2012; TCGA, unpublished
**D34A/Y**	2	D75	UCH	Inserm[h], 2015; MSKCC, 2017
**L35F**	1	L76	UCH	DFCI, 2015
**G41C**	1	S82	UCH	MSKCC, 2017
**V43A**	1	P83	UCH	DFCI, 2016
**G45E/R**	2	G85	UCH	TCGA, unpublished
**I47M**	2	I87	UCH	TCGA, 2014; MSKCC, 2017; TCGA, unpublished
**L49R/V/P**	4	L89	UCH	METABRIC, 2012; TCGA, 2012; TCGA, 2013; TCGA, 2015; METABRIC, 2016; TCGA, unpublished
**K51I**	1	R91	UCH	U Tokyo, 2013

Mutation ID in BAP1	# Instances	Corresponding residue in Calypso	Domain ID	References
**E54K**	1	E94	UCH	TCGA, 2013
**R56C**	3	R96	UCH	METABRIC, 2012; U Tokyo, 2014; METABRIC, 2016; MSKCC, 2017
**R57Q**	1	R97	UCH	MSKCC, 2017
**R59Q/W**	3	R99	UCH	MSKCC, 2017; TCGA, unpublished
**R60Q**	2	R100	UCH	Novartis/Broad, 2012
**S63C**	1	V103	UCH	MSKCC, 2017
**D67G**	1	A107	UCH	MSKCC, 2017
**D68G**	2	E108	UCH	MSKCC, 2017; TCGA, unpublished
**V71M**	1	V111	UCH	MSKCC, 2017
**I72T/S**	4	K112	UCH	MSK, 2017; MSKCC, 2017
**D73Y**	1	D113	UCH	JHU, 2014
**D75G**	1	E115	UCH	TCGA, 2013; TCGA, unpublished
**I76T**	1	A116	UCH	TCGA, unpublished
**N78S**	4	S118	UCH	TCGA, 2013; Singapore, 2014; TCGA, unpublished
**N79S**	1	S119	UCH	Novartis/Broad, 2012
**F81L**	1	F121	UCH	MSKCC, 2017
**Q85H**	1	Q125	UCH	NYU[i], 2015
**S90C**	1	S130	UCH	MSKCC, 2017
**C91G/F/R/Y**	6	C131	UCH (active site)	U Tokyo, 2013; NYU, 2015; MSKCC, 2017
**T93A**	1	T133	UCH	TCGA, 2015; TCGA, unpublished
**H94D/R**	2	H134	UCH	MSKCC, 2017
**A95D/T**	2	A135	UCH	Novartis/Broad, 2012; Robinson *et al.*, 2015
**V99M**	3	V139	UCH	TCGA, 2014; MSKCC, 2017; TCGA, unpublished
**N102I/D/S**	3	N142	UCH	METABRIC, 2012; UCLA[j], 2014; METABRIC, 2016; MSKCC, 2017
**S105I**	1	E145	UCH	METABRIC, 2012; METABRIC, 2016

Mutation ID in BAP1	# Instances	Corresponding residue in Calypso	Domain ID	References
**G109V**	2	G151	UCH	TCGA, 2013; MSK, 2017; MSKCC, 2017; TCGA, unpublished
**R114C**	1	R156	UCH	MSKCC, 2017
**F118V**	1	H160	UCH	TCGA, unpublished
**K120E**	2	K162	UCH	METABRIC, 2012; METABRIC, 2016; MSK, 2017
**S123T**	1	S165	UCH	Broad, 2013
**E125D/K**	2	E167	UCH	MSKCC, 2017
**G132D**	1	G174	UCH	MSKCC, 2017
**P135R/L**	3	P177	UCH	Broad, 2012; TCGA, 2016; MSK, 2017; MSKCC, 2017
**A138T/V**	2	A180	UCH	TCGA, 2015; MSKCC, 2017 TCGA, unpublished
**A140V**	2	A182	UCH (crossover-loop)	Broad, 2012; TCGA, 2016
**H141N/Y**	3	H183	UCH (crossover-loop)	MSKCC, 2017
**S143N**	1	S185	UCH (crossover-loop)	TCGA, unpublished
**H144D**	1	H186	UCH (crossover-loop)	MSK, 2017; MSKCC, 2017
**A145G**	1	A187	UCH (crossover-loop)	TCGA, 2013; TCGA, unpublished
**R146K**	2	M188	UCH (crossover-loop)	MSKCC, 2017
**P149Q**	1	A191	UCH (crossover-loop)	MSKCC, 2016
**R150H**	1	R192	UCH (crossover-loop)	DFCI, 2016
**G158D**	1	G201	UCH (crossover-loop)	MSK, 2018
**R163W/Q**	2	F207	UCH (crossover-loop)	TCGA, 2014; DFCI, 2016; TCGA, 2017; TCGA, unpublished
**H169Y/Q**	5	H213	UCH (active site and crossover-loop)	BGI, 2013; JHU, 2013; Singapore, 2014; MSKCC, 2017; TCGA, unpublished

Mutation ID in BAP1	# Instances	Corresponding residue in Calypso	Domain ID	References
**F170V/C/L**	5	F214	UCH (crossover-loop)	Broad/Cornell, 2012; TCGA, 2013; MSKCC, 2017; TCGA, unpublished
**V171D**	1	V215	UCH	BGI, 2012
**S172N/I**	3	S216	UCH	MSKCC, 2017
**Y173C/S**	6	F217	UCH	MSK, 2017; MSKCC, 2017; TCGA, unpublished
**P175S/H**	3	P219	UCH	Novartis/Broad, 2012; TCGA, 2014; MSKCC, 2017; TCGA, unpublished
**G178V**	1	G222	UCH	Novartis/Broad, 2012
**R179W**	3	Q223	UCH	METABRIC, 2012; TCGA, 2014; METABRIC, 2016; QCMG[k], 2016; TCGA, 2016; TCGA, unpublished
**E182D**	1	E226	UCH	MSKCC, 2017
**D184A/H**	2	D228	UCH (active site)	MSKCC, 2014; MSKCC, 2017
**G185R**	10	G229	UCH	IRC[l], 2014; MSKCC, 2017; TCGA, unpublished
**L186Q**	1	L230	UCH	MSKCC, 2017
**Y189F**	1	Y233	UCH	MSKCC, 2017
**P190H**	2	P234	UCH	TCGA, 2014; MSKCC, 2017; TCGA, unpublished
**G194R**	2	G238	UCH	MSK, 2016; TCGA, unpublished
**W196G**	1	W240	UCH	TCGA, unpublished
**E200G**	1	E244	UCH	TCGA, 2012
**W202L**	1	W246	UCH	TCGA, unpublished
**T203A**	1	T247	UCH	METABRIC, 2012; METABRIC, 2016
**R208Q/W/G**	4	R252	UCH	Novartis/Broad, 2012; MSKCC, 2017
**I210T**	1	M254	UCH	MSKCC, 2017
**M211V**	1	A255	UCH	TCGA, 2013; TCGA, unpublished
**R213H**	1	R257	UCH	MSKCC, 2017
**I214M**	1	L258	UCH	TCGA, unpublished
**A217T**	1	A261	UCH	MSKCC, 2017
**E221K**	2	-	UCH	DFCI/MSKCC, 2014 MSKCC, 2017

Mutation ID in BAP1	# Instances	Corresponding residue in Calypso	Domain ID	References
**P222T**	1	-	UCH	METABRIC, 2012; METABRIC, 2016
**Y223F**	1	-	UCH	MSK, 2017; MSKCC, 2017
**D225N/H/Y**	6	D266	UCH	Sanger, 2012; MSK, 2013; TCGA, 2014; MSKCC, 2017; TCGA, unpublished
**R227H/C/P/L**	13	R268	UCH	TCGA, 2011; TCGA, 2012; MSK, 2013; TCGA, 2016; MSK, 2017; MSKCC, 2017; TCGA, unpublished
**N229H**	1	N270	UCH	TCGA, 2013; TCGA, unpublished
**L230Q**	1	L271	UCH	U Tokyo, 2013
**M231T/I/K**	3	M272	UCH	BGI, 2013; MSKCC, 2017
**R237C/H**	2	R278	ULD	Broad, 2013; TCGA, 2015; DFCI, 2016
**E242G/K**	2	T283	ULD	TCGA, 2013; MSKCC, 2017
**L245P**	1	L286	ULD	TCGA, 2014; TCGA, unpublished
**R252P**	1	Q293	ULD	METABRIC, 2012; METABRIC, 2016
**I263M**	1	L303	BAP1/Calypso insertion	Novartis/Broad, 2012
**R264T**	1	-	BAP1/Calypso insertion	METABRIC, 2012; METABRIC, 2016
**T266P**	1	K305	BAP1/Calypso insertion	TCGA, unpublished
**I271T**	2	-	BAP1/Calypso insertion	MSK, 2016; TCGA, unpublished
**N290S**	1	Q318	BAP1/Calypso insertion	MSKCC, 2017
**S292F**	1	-	BAP1/Calypso insertion	MSK, 2017
**P293L/S**	4	P320	BAP1/Calypso insertion	METABRIC, 2012; METABRIC, 2016; MSK, 2017; MSKCC, 2017
**N299S**	1	-	BAP1/Calypso insertion	TCGA, 2012; TCGA, unpublished

Mutation ID in BAP1	# Instances	Corresponding residue in Calypso	Domain ID	References
**A303P**	1	-	BAP1/Calypso insertion	MSKCC, 2013
**G307D**	1	-	BAP1/Calypso insertion	Novartis/Broad, 2012
**D311Y**	1	-	BAP1/Calypso insertion	TCGA, unpublished
**G312C**	1	-	BAP1/Calypso insertion	Michigan, 2012
**A316V**	1	-	BAP1/Calypso insertion	DFCI, 2016
**A321V/T**	3	-	BAP1/Calypso insertion	METABRIC, 2012; METABRIC, 2016; MSKCC, 2017
**A323S**	1	-	BAP1/Calypso insertion	TCGA, unpublished
**S325F**	2	-	BAP1/Calypso insertion	MSK, 2017; MSKCC, 2017
**P328S**	1	-	BAP1/Calypso insertion	MSKCC, 2017
**K331E**	1	-	BAP1/Calypso insertion	METABRIC, 2012; METABRIC, 2016
**P332L**	1	-	BAP1/Calypso insertion	MSKCC, 2014
**P338S**	1	-	BAP1/Calypso insertion	TCGA, 2014; TCGA, unpublished
**G340S**	1	-	BAP1/Calypso insertion	TCGA, 2016
**N344S**	1	-	BAP1/Calypso insertion	Broad, 2013
**G345V**	1	-	BAP1/Calypso insertion	MSKCC, 2017
**V354I/G**	3	T325	BAP1/Calypso insertion	METABRIC, 2012; METABRIC, 2016; MSKCC, 2017
**L357M**	1	E328	BAP1/Calypso insertion	TCGA, 2013; TCGA, unpublished
**L361V**	2	F332	BAP1/Calypso insertion	Broad, 2012; TCGA, 2016
**P370H**	1	-	BAP1/Calypso insertion	TCGA, unpublished
**Q372E**	1	-	BAP1/Calypso insertion	METABRIC, 2012; METABRIC, 2016
**E374Q**	1	-	BAP1/Calypso insertion	Roger, 2016; UCLA, 2016
**A379T**	1	-	BAP1/Calypso insertion	MSKCC, 2017
**R383H**	1	-	BAP1/Calypso insertion	Novartis/Broad, 2012

Mutation ID in BAP1	# Instances	Corresponding residue in Calypso	Domain ID	References
**R385Q**	1	-	BAP1/Calypso insertion	JHU, 2014
**E398D**	1	-	BAP1/Calypso insertion	MSKCC, 2017
**D399N**	2	-	BAP1/Calypso insertion	TCGA, 2013; TCGA, 2015; TCGA, unpublished
**Y401D**	1	-	BAP1/Calypso insertion	Novartis/Broad, 2012
**D404E**	1	-	BAP1/Calypso insertion	METABRIC, 2012; METABRIC, 2016
**V409L/A**	2	-	BAP1/Calypso insertion	TCGA, 2013 MSKCC, 2017; TCGA, unpublished
**N413S**	1	-	BAP1/Calypso insertion	MSKCC, 2017
**L416F**	1	-	BAP1/Calypso insertion	TCGA, unpublished
**R417M**	2	-	BAP1/Calypso insertion	Novartis/Broad, 2012; TCGA, unpublished
**G422R**	1	-	BAP1/Calypso insertion	METABRIC, 2012; METABRIC, 2016
**L429W**	1	-	BAP1/Calypso insertion	Robinson *et al.*, 2015
**L437P**	1	-	BAP1/Calypso insertion	Novartis/Broad, 2012
**V439L**	3	-	BAP1/Calypso insertion	JHU, 2013; MSKCC, 2017; TCGA, unpublished
**N446S**	1	-	BAP1/Calypso insertion	METABRIC, 2012; METABRIC, 2016
**V447I**	2	-	BAP1/Calypso insertion	METABRIC, 2012; Novartis/Broad, 2012; METABRIC, 2016
**E450K**	1	-	BAP1/Calypso insertion	MSK, 2017; MSKCC, 2017
**K457R**	1	-	BAP1/Calypso insertion	TCGA, unpublished
**P462S**	1	-	BAP1/Calypso insertion	MSKCC, 2017
**L463V**	1	-	BAP1/Calypso insertion	Novartis/Broad, 2012
**I465M**	1	-	BAP1/Calypso insertion	MSK, 2017; MSKCC, 2017
**G472R**	1	-	BAP1/Calypso insertion	MSKCC, 2017

Mutation ID in BAP1	# Instances	Corresponding residue in Calypso	Domain ID	References
**S482L**	1	-	BAP1/Calypso insertion	Novartis/Broad, 2012
**P486L**	1	-	BAP1/Calypso insertion	MSKCC, 2017
**N490S**	1	-	BAP1/Calypso insertion	MSKCC, 2017
**S492N**	1	-	BAP1/Calypso insertion	MSKCC, 2017
**T495M**	1	-	BAP1/Calypso insertion	TCGA, 2013; TCGA, unpublished
**A496T**	1	-	BAP1/Calypso insertion	TCGA, 2013; TCGA, unpublished
**E498K/Q**	4	-	BAP1/Calypso insertion	MSK, 2017; MSKCC, 2017
**G500D**	2	-	BAP1/Calypso insertion	Novartis/Broad, 2012; DFCI, 2016
**A502P**	1	-	BAP1/Calypso insertion	TCGA, unpublished
**R508C**	1	-	BAP1/Calypso insertion	Novartis/Broad, 2012
**R512L/C**	3	-	BAP1/Calypso insertion	Novartis/Broad, 2012; TCGA, 2012; DFCI, 2016; TCGA, unpublished
**P516L**	1	-	BAP1/Calypso insertion	Yale, 2012
**T517M**	4	-	BAP1/Calypso insertion	NCI[m], 2012; Novartis/Broad, 2012; Institut Curie, 2014; MSKCC, 2015
**R518L**	1	-	BAP1/Calypso insertion	MSKCC, 2017
**P519L**	1	-	BAP1/Calypso insertion	MSKCC, 2017
**P522T**	1	-	BAP1/Calypso insertion	Novartis/Broad, 2012
**V523I**	1	-	BAP1/Calypso insertion	Novartis/Broad, 2012
**S525C**	1	-	BAP1/Calypso insertion	BCCRC[n], 2012
**H526Y**	1	-	BAP1/Calypso insertion	MSKCC, 2017
**L539Q**	1	-	BAP1/Calypso insertion	TCGA, unpublished
**R540H**	1	-	BAP1/Calypso insertion	CPC GENE[o], 2017

**Mutation ID in BAP1**	**# Instances**	**Corresponding residue in Calypso**	**Domain ID**	**References**
**V541G**	1	-	BAP1/Calypso insertion	TCGA, 2014; TCGA, unpublished
**Y546H**	1	-	BAP1/Calypso insertion	TCGA, unpublished
**R548C**	2	-	BAP1/Calypso insertion	TCGA, 2013; TCGA, unpublished
**P555T**	1	-	BAP1/Calypso insertion	METABRIC, 2012; METABRIC, 2016
**E566K**	2	-	BAP1/Calypso insertion	METABRIC, 2012; TCGA, 2015; METABRIC, 2016; TCGA, unpublished
**A574V**	1	-	BAP1/Calypso insertion	MSK, 2017 MSKCC, 2017
**G579R**	1	-	BAP1/Calypso insertion	MSKCC, 2014
**I586L**	2	-	BAP1/Calypso insertion	Broad, 2011; TCGA, 2015; TCGA, unpublished
**P588T**	1	-	BAP1/Calypso insertion	Broad, 2013
**K601E**	1	-	BAP1/Calypso insertion	METABRIC, 2012; METABRIC, 2016
**E602V**	1	-	BAP1/Calypso insertion	METABRIC, 2012; METABRIC, 2016
**V604M**	1	-	BAP1/Calypso insertion	TCGA, 2014; TCGA, unpublished
**T607M**	2	-	BAP1/Calypso insertion	Broad, 2014; DFCI, 2016
**R610K**	1	-	BAP1/Calypso insertion	MSKCC, 2017
**K612M**	1	-	BAP1/Calypso insertion	Broad, 2012
**T613M**	1	-	BAP1/Calypso insertion	MSK, 2017; MSKCC, 2017
**G619D**	1	-	BAP1/Calypso insertion	TCGA, 2013; TCGA, unpublished
**L622M**	1	-	BAP1/Calypso insertion	TCGA, 2014; TCGA, 2016; TCGA, unpublished
**P629S**	1	-	BAP1/Calypso insertion	TCGA, unpublished
**E631Q/V**	4	-	BAP1/Calypso insertion	TCGA, 2014; MSK, 2017; MSKCC, 2017; TCGA, unpublished

Mutation ID in BAP1	# Instances	Corresponding residue in Calypso	Domain ID	References
**L632P**	1	-	BAP1/Calypso insertion	TCGA, 2016
**V639L**	1	L344	ULD	NCI, 2012
**A641T**	1	T346	ULD	MSKCC, 2017
**E642D**	1	E347	ULD	MSKCC, 2017
**I643T**	1	I348	ULD	MSKCC, 2017
**A644V**	1	A349	ULD	Novartis/Broad, 2012
**C649Y**	1	H354	ULD	METABRIC, 2012; METABRIC, 2016
**E653K**	1	E358	ULD	MSKCC, 2017
**K656N**	1	R361	ULD	TCGA, 2014; TCGA, unpublished
**R657W**	1	R362	ULD	MSKCC, 2017
**K658N**	1	H363	ULD	MSKCC, 2014
**I662N**	1	V367	ULD	TCGA, 2013
**D663H**	1	D368	ULD	TCGA, 2014; TCGA, unpublished
**R667K**	1	R372	ULD	MSKCC, 2017
**I675F**	1	I380	ULD	MSKCC, 2017
**F678L**	1	F383	ULD	MSKCC, 2017
**E685V**	1	Q390	ULD	TCGA, unpublished
**G686C**	1	G391	ULD	TCGA, 2013; TCGA, unpublished
**I696T**	3	L401	ULD	MSKCC, 2017
**R699P/W**	3	S404	ULD	METABRIC, 2012; METABRIC, 2016; MSKCC, 2017
**R700Q**	1	K405	ULD	TCGA, 2013; TCGA, unpublished
**R701C**	1	K406	ULD	MSKCC, 2017
**R717Q/W**	3	G458	ULD (C-ter. tail)	METABRIC, 2012; DFCI, 2016; METABRIC, 2016; MSKCC, 2017
**R718W**	1	R459	ULD (C-ter. tail)	MSKCC, 2017
**P723A/S**	3	C467	ULD (C-ter. tail)	MSKCC, 2014; MSK, 2017

Mutation ID in BAP1	# Instances	Corresponding residue in Calypso	Domain ID	References
**K727M**	1	-	ULD (C-ter. tail)	MSKCC, 2017
**R728H**	1	-	ULD (C-ter. tail)	MSKCC, 2017

[a]TCGA, The Cancer Genome Atlas. [b]DFCI, Dana-Farber Cancer Institute. [c]JHU, Johns Hopkins University. [d]MSK/MSKCC, Memorial Sloan Kettering Cancer Center. [e]BGI, Beijing Genomics Institute. [f]METABRIC, Molecular Taxonomy of Breast Cancer International Consortium. [g]U Tokyo, University of Tokyo. [h]Inserm, National Institute of Health and Medical Research. [i]NYU, New York University. [j]UCLA, University of California, Los Angeles. [k]QCMG, Queensland Centre for Medical Genomics. [l]IRC, Institute of Cancer Research. [m]NCI = National Cancer Institute. [n]BCCRC, BC Cancer Research Centre. [o]CPC GENE, Canadian Prostate Cancer Genome Network.

Supplementary Table 3. Cancer-derived missense mutations in the NEF-motifs of ASXL1 and ASXL2

Mutation ID in ASXL1	# Instances	Corresponding residue in ASX	References
**H315R/N/Y**	3	R288	TCGA[a], 2012; MSKCC[b], 2017; MSK[b], 2018
**Mutation ID in ASXL2**	**# Instances**	**Corresponding residue in ASX**	**References**
**N329K**	1	N283	MSKCC, 2017
**E330K/Q**	7	E284	METABRIC[c], 2012; TCGA, 2014 METABRIC, 2016; MSKCC, 2017; TCGA, unpublished
**F331L**	2	F285	TCGA, 2012; TCGA, 2013; TCGA, unpublished
**S334L**	1	R288	TCGA, 2015; TCGA, unpublished

[a]TCGA, The Cancer Genome Atlas. [b]MSK/MSKCC, Memorial Sloan Kettering Cancer Center. [c]METABRIC, Molecular Taxonomy of Breast Cancer International Consortium.

Supplementary Table 4. Crystallographic interfaces identified by PISA in the Calypso–ASX structure

**Interface #**	**Chain ID** (Structure 1)	**Domain ID** (Structure 1)	**Symmetry op.** (Structure 2)	**Chain ID** (Structure 2)	**Domain ID** (Structure 2)	**Interface area** (Å)	**ΔG** (kcal/mol)	**ΔG** P-value
**1**	D	ASX DEU_B	x, y, z	C	Calypso UCH/ULD_B	1420.6	-26.4	0.091
**2**	B	ASX DEU_A	x, y, z	A	Calypso UCH/ULD_A	1350.1	-23.5	0.111
**3**	A	Calypso UCH/ULD_A	-x, y, -z	A	Calypso UCH/ULD_A	599.4	-2.6	0.604
**4**	C	Calypso UCH/ULD_B	-x+1/2, y-1/2, -z+1/2	B	ASX DEU_A	517.6	-2.6	0.701
**5**	C	Calypso UCH/ULD_B	x, y, z	A	Calypso UCH/ULD_A	399.5	-8.3	0.085

Supplementary Table 5. SAXS data collection and analysis statistics

	Calypso–ASX complex
**Data-collection parameters**	
Instrument	Australian Synchrotron SAXS/WAXS beamline
Beam geometry	120 μm point source
Wavelength (Å)	1.033
Exposure time	2 sec exposures
Temperature (K)	285
q range ($Å^{-1}$)[a]	0.00644 to 0.250
Protein concentration	60 µL of 8 mg/ml protein *via* inline gel filtration chromatography in 0.5M NaCl, 20mM HEPES pH 7.5, 5% v/v glycerol, 0.2mM TCEP
**Structural parameters**	
I(0) (cm^{-1}) [from P(r)]	0.02506 ± 0.0001
R_g (Å) [from P(r)]	40.60 ± 0.24
D_{max} (Å)	125
I(0) (cm^{-1}) (from Guinier)	0.02505 ± 0.0002
R_g (Å) (from Guinier)	40.40 ± 0.49
**Software employed**	
Primary data reduction	Scatterbrain (Australian Synchrotron)
Data processing	PRIMUS, GNOM
Computation of model intensities	CRYSOL

[a]q is the magnitude of the scattering vector, which is related to the scattering angle (2θ) and the wavelength (λ) as follows: $q = (4\pi/\lambda)\sin\theta$

SUPPLEMENTARY REFERENCES

1. Sahtoe, D.D. et al. Mechanism of UCH-L5 activation and inhibition by DEUBAD domains in RPN13 and INO80G. *Mol Cell* **57**, 887-900 (2015).

2. Krissinel, E. & Henrick, K. Inference of macromolecular assemblies from crystalline state. *J Mol Biol* **372**, 774-97 (2007).
